# The Effectiveness of Group Assertiveness Training on Happiness in Rural Adolescent Females with Substance Abusing Parents

**DOI:** 10.5539/gjhs.v8n2p156

**Published:** 2015-06-11

**Authors:** Seyed Kaveh Hojjat, Ebrahim Golmakani, Mina Norozi Khalili, Maryam Shakeri Chenarani, Mahin Hamidi, Arash Akaberi, Amir Rezaei Ardani

**Affiliations:** 1 Addiction and Behavioral Sciences Research Center, North Khorasan University of Medical Sciences, Bojnurd, Iran; 2 Departments of Anesthesiology, Faculty of Medicine, Mashhad University of Medical Sciences, Mashhad, Iran; 3 Department of Community Medicine, School of Medicine, North Khorasan University of Medical Sciences, Bojnurd, Iran; 4 Islamic Azad University, Ghuchan Branch, ghuchan, Iran; 5 Department of Epidemiology and Biostatistics, Sabzevar University of Medical Sciences, Sabzevar, Iran; 6 School of Continuing Studies, McGill University, Montreal, QC, Canada; 7 Psychiatry and Behavioral Sciences Research Center, Mashhad University of Medical Sciences, Mashhad, Iran

**Keywords:** substance abusing parents, assertiveness training, happiness, rural, adolescents

## Abstract

**Background:**

Parental substance abuse confronts children with a variety of psychological, social, and behavioral problems. Children of substance abusing parents show higher levels of psychiatric disorders including anxiety and depression and exert lower levels of communication skills. Weak social skills in this group of adolescents put them at a higher risk for substance abuse. Many studies showed school based interventions such as life skill training can effective on future substance abusing in these high risk adolescences.

**Materials and Methods:**

The participants consisted of 57 middles schools girls, all living in rural areas and having both parents with substance dependency. The participants were randomly assigned to intervention (n=28) and control (n=29) groups. The data were collected before and six weeks after training in both group. The intervention group received eight sessions of group assertiveness training. Participants were compared in terms of changes in scores on the Oxford Happiness Questionnaire and the Gambrills-Richey Assertion Inventory.

**Results:**

The total score for happiness change from 43.68 ±17.62 to 51.57 ±16.35 and assertiveness score changed from 110.33±16.05 to 90.40±12.84. There was a significant difference in pretest-posttest change in scores for intervention (7.89±4.13) and control (-2.51±2.64) groups; t (55) =2.15, p = 0.049. These results suggest that intervention really does have an effect on happiness and assertiveness.

**Conclusion:**

Determining the effectiveness of these school based interventions on other life aspects such as substance abuse calls for further study on these rural adolescent girls.

## 1. Introduction

It has been found that about 10% of adolescent live with parents who actively abuses alcohol or illicit drug (Donohue, Romero, & Hill, 2006). According to the 2008 report by the Substance Abuse and Mental Health Services Administration (SAMHA), over 19 million children are living with at least one alcohol or substance abusing parent in the United States alone (Aldworth, 2009). Negative effects of parental substance abuse documented by many studies include physical, psychological and cognitive consequence for children development (Donohue et al., 2006; Kilpatrick et al., 2000; Reinherz, Giaconia, Hauf, Wasserman, & Paradis, 2000). Substance abusing parents expose their children to the risk of substance abuse and other behavioral problems (Ashenberg Straussner & Huff Fewell, 2012). Children of substance abusing parents are at risk for lifelong problems, such as low self-esteem, interpersonal problems and feelings of isolation (Anda et al., 2006).

The adolescents, although less susceptible than younger children to being physically harmed by their parents, but they have more extended exposure to their substance user parents and its outcomes (Peleg-Oren & Teichman, 2006). The adolescents with drug user parents than other peers have been showed to have higher risk of negative self-image and feeling of lonely and less communication skills such as planning, stress management and impulse control (McGrath, Watson, & Chassin, 1999; Perez-Bouchard, Johnson, & Ahrens, 1993).

Happiness is defined as achieving whatever you want, and well-being is sometimes considered synonymous to happiness. Happiness is generally considered to comprise three main components: the frequency and degree of positive affect and joy; the absence of negative feelings, such as depression or anxiety; and the average level of satisfaction over a period (Stewart, Watson, Clark, Ebmeier, & Deary, 2010). Psychologists, as argyle, said that happiness is a combination of existing positive feeling, nonexistence of negative emotions and life satisfaction (M Argyle & Lu, 2001).The people who are happy have more feeling of safety, decide easier and have more participatory temperament and much more sense of satisfaction with who live along them (Myers, 2001). Optimism and positive are effective in dealing with life’s pressures, overcoming psychosocial difficulties, displaying health-related behaviors, modifying lifestyle, and finally in reducing the occurrence of physical and psychological illnesses (Posadzki, Stockl, Musonda, & Tsouroufli, 2010).

During adolescence, they see everything into crisis, have mood fluctuation, be impulsive and sensitive, and they are not able to projecting. The Adolescents with drug addicted parents have more likely to confront a complex of stress factors within the family and their own environment. The adolescents with addicted family may believe that they have not power for change or improve their lives (Harter, 2000). This can lead to lower levels of happiness and life satisfaction in these children compared to others (Figlie, Fontes, Moraes, & Payá, 2004; Jayasvasti & Kanchanatawan, 2005). Adolescents often feel of fault and shame for their addicted parents and avoid discussing with peers, authorities and helping people. In early adolescents (ages 10-15), many children begin experimenting and using alcohol and other drugs (Emshoff & Price, 1999).

Assertiveness is an individual ability that has received much attention in the past two decades. (Townend, 2007) The psychological concept of assertiveness covers three major principles of human expression: behavior, cognition, and emotion. From the behavioral aspect, assertiveness is the skill that enables a person to freely express his feelings, defend his objectives and goals in ordinary and special circumstances, and have a sense of accomplishment and success in interpersonal relationships (Townend, 2007). In terms of affectivity, assertive individuals are able to express and respond to their positive and negative feelings without anxiety and unnecessary anger (Townend, 2007). Researchers have indicated that training skills on problem solving, stress management, assertiveness, anger management and emotional self-awareness bring about higher levels of happiness in individuals (Cohn, Fredrickson, Brown, Mikels, & Conway, 2009).

Until the past few years, the issue of drug abuse in rural polities had low priority to research (I. C. Rhew, J. David Hawkins, & S. Oesterle, 2011). Despite previous studies that drug use was more prevalent among resident of urban, the prevalence of drug use among rural-dwelling now is convergent or even has transcended than urban resident (Cronk & Sarvela, 1997; Gfroerer, Larson, & Colliver, 2007; Isaac C. Rhew, J. David Hawkins, & Sabrina Oesterle, 2011). Young adults living in rural-large areas have higher rate of substance abuse than their urban peers. Residents of rural area are more likely than urban to have risky behavior affected by substance use (Isaac C. Rhew et al., 2011). The problem of drug use may be additive among rural residential settings, because of reduced access to treatment services due to demographic characteristics and geographic context of rural settings (DeVoe, Krois, & Stenger, 2009).

To date, there have been few major studies evaluating the effectiveness of clinical interventions specifically for adolescents with substance-involved parents. Group treatment is a common intervention for adolescent with substance abuser parents (Ashenberg Straussner & Huff Fewell, 2012). A number of evidence- based family programs are used, such as strengthening families program (SFP). The SFP is a family skills training program planned for increase resilience and reduce risk factors for behavioral, emotional, academic and social problems in families with addicted parents (Ashenberg Straussner & Huff Fewell, 2012).

Considering complexity and display of psychological and behavioral problems among children and adolescents, the necessity to learning of life skills and practical solution in this group feels any more than the others. For these reasons and into the goal of early and effective intervention, a trial for effectiveness of school-based services affording psychological education are targeted to adolescents in this study Therefore, in this research aimed to evaluate the effect of group assertiveness training on assertiveness and happiness of rural adolescents.

## 2. Objectives

The objective of this study was to assess the effects of assertiveness skill training on happiness and self-assertiveness on rural girls with drug dependents parents.

## 3. Materials & Methods

This investigation was a pretest-posttest experimental research with control group. Prior to the study’s commencement, the researchers asked approval to conduct the study from the principal of the middle school. Participants were from four nearby villages around the city of Bojnurd, Northeastern Iran, with a population of about 5000 persons in year 2014. The subjects were selected among middle school girls in Jannat School if inclusion and exclusion criteria were met. The inclusion criteria were as follows:(1) female gender and unmarried; (2) 12-15 years old; (3) living in rural areas; (4) having both parents with opium dependency (substance dependence in parents was confirmed through the students’ initial report to the school counselor and parents’ own report on opium dependency); (5) parents and students consent for taking part in the study; (6) not having an immediate family member who has died in the past six months (due to its impact on happiness levels); (7) having no history of major psychiatric disorders (based on an interview with a psychiatrist). The exclusion criteria were (1) Absence from more than three group therapy sessions; (2) occurrence of unfortunate happenings in the course of treatment (such as the death of close relatives). To begin with, samples participated in an orientation session to become familiar with the research process. Finally, sixty students were recruited in our study. In the first stage, the subjects were randomly divided into two groups, with 30 student in experimental group and 30 in the control group. For random allocation we used computer generated random numbers. All participants filled self-assertiveness and happiness questionnaires as pretest. Then, intervention group received group assertiveness training. The training sessions included eight sessions twice a week. The group’s emphasis was on role-play. In each session, six group members participated in role-play and by the end of all sessions; every group member should have been participated in this activity. Due to ethical considerations, the control group was given an explanation that the interventions will be implemented for them as well at the end of the control group period. Six week after finishing training, both groups (control and intervention) answered the questionnaires at the same conditions again as posttest. All participants and their parents provided written consent stating their willingness to participate in this study.

## 3.1 Measures

### 3.1.1 The Oxford Happiness Questionnaire

This 29-item questionnaire was devised by Argyle in 1990. This questionnaire has been prepared by using a Likert scale. Argyle et al. reported an internal reliability (Cronbach’s alpha) of 0.90 for this questionnaire (Hills & Argyle, 2002). The Persian versions of this questionnaire were evaluated by Alipor in 2000. Alipor reported an internal reliability (Cronbach’s alpha) of 0.94 in men and 0.90 in women (Liaghatdar, Jafari, Abedi, & Samiee, 2008). Participants determined their happiness as the answer to the four optional questions (as the range of 0= I am completely agreeable to 3= I am completely opposite).

### 3.1.2 The Gambrill-Richey Assertion Inventory

This 40-item scale was devised by Gambrill and Richey in 1975. This questionnaire has been prepared in Likert scale. The participants determined their self-assertion as “degree of discomfort” and “probability of engaging in assertive behavior” (Gambrill & Richey, 1975). This scale includes two sections, one measures discomfort, and other is for the measurement of daringly behaviors. Gambrill and Richey reported an internal reliability of 0.81 for this questionnaire. The gradation of this scale lines as “I trouble none, I trouble a little, I trouble dubious, I trouble high, and I trouble greatly high” for each sentence. The validity of this scale is between 0.3 and 0.7, and Reliability coefficient is 0.81. Also, validity and reliability of questionnaire were checked among Iranian samples (Ilkhchi, Poursharifi, & Alilo, 2011)

### 3.2 Statistical Analysis

In this study, the quantitative variables of the two groups were compared using chi square-tests. Within the groups, paired t-test was used to compare differences between before and after training. The Independent t-test was used to compare the means of quantitative variables from the two groups. Normality of variables assessed by Levene’s Test and homogeneity of Variances in all variables confirmed (p>0.05). The level of significance in this study was set at 0.05. Description and analysis of data were carried out using the SPSS 16 software.

## 4. Results

By the end of the research, two participants from the intervention group and one participant from the control group dropped out of the study. (One girl in each group married and leaved the school and one girl changed her school). Thus, 57 individuals were evaluated in the two groups: 28 in the intervention group and 29 in the control group .mean age of participant in intervention group were 14.1±0.4 and 14.4±0.25 in control group. An independent-samples t-test was conducted to compare mean age and not significant difference was seen t(55)=0.36, p=0.44. The fathers and mothers of the girls under study also showed no significant difference in terms of age, educational status, and employment (Table 1).

**Table 1:** Demographic features of parents in intervention and control group. (Include dropped out cases)

		Group		

		intervention	Control	p-value
Father’s age (Mean±SD)		43.2±8.6	48.4±9.45	0.126
Mother’s age (Mean±SD)		39.73±6.75	41.31±6.6	0.515
Father’s education	Illiterate	6(20.0%)	8(26.6%)	0.285^F^
	Primary school	18(60.0%)	8(26.6%)	
	Guidance school	4(13.3%)	10(33.3%)	
	High school	2(6.7%)	4(13.3%)	
Mother’s education	Illiterate	12(40.0%)	6(20.0%)	0.412^F^
	Primary school	14(46.7%)	20(66.6%)	
	Guidance school	4(13.3%)	4(13.3%)	
Father’s occupation	Labor	16(53.3%)	16(53.3%)	0.802^F^
	Farmer	6(20.0%)	7(23.3%)	
	Others	8(26.7%)	7(23.3%)	
Mother’s occupation	Employed	0)0.0 %(	2) 6.7 %(	1 ^F^
	Housekeeper	30)100.0 %(	28) 93.3 %(	

F:*P-value according Fisher’s exact test.*

The mean assertiveness score in intervention group was 110.33±16.5 and 107.68±16.71 in control group and was not significantly different in the pretest stage (p=0.65).Also the mean score for total happiness in the pretest stage was not significantly different (p=0.917). In addition, the two groups showed not statistically significant difference in the pretest stage regarding components of happiness (subjective well-being, life satisfaction, respect, satisfaction) (Table 2).we used pair sample t test for compare pretest-posttest change in each group. There was a significant difference in pretest-posttest change in scores of assertiveness in intervention group t (27) =7.25, p<0.00001, but this change was not significant in control group. t (28) =0.36, p=0.72. Also there was a significant difference in pretest-posttest change in scores of oxford happiness in intervention group t (27) =1.98, p=0.042, but this change was not significant in control group .t (28) =0.95, p=0.35.

**Table 2:** Baseline data in the intervention (n=28) and control (n=29) groups

Group			
Oxford happiness subscales	intervention	Control	P value
Total score of happiness	43.68±17.62	44.25 ±12.39	0.10
subjective well-being	35.93±18.77	37.84±14.93	0.75
life satisfaction	40.28± 26.10	42.44±16.33	0.78
respectability	42.54±15.22	41.07±13.78	0.78
contentment	48.15±25.55	47.91±20.97	0.97
Total score of assertiveness	110.33±16.05	107.68±16.71	0.65

An independent-samples t-test was conducted to compare total oxford happiness score for intervention and control groups. There was a significant difference in pretest-posttest change in scores for intervention (7.89±4.13) and control (-2.51±2.64) groups; t (55) =2.15, p = 0.049. These results suggest that intervention really does have an effect on happiness. Also an independent-samples t-test was conducted to compare Gambrills-Richey assertiveness score for intervention and control groups. There was a significant difference in pretest-posttest change in scores for intervention (-19.93) and control (-0.75) groups; t (55) =5.63, p < 0.0001. These results suggest that intervention really does have an effect on assertiveness. Independent-samples t-test also was conducted to compare components of oxford happiness scale. Results showed a significant difference on subjective well-being. t(55) =5.23, p=0.02 but there was not significant in life satisfaction (p=0.23) and respectability (p=0.59) and Contentment(p=0.09). The results are summarized in Table 3.

**Table 3:** Changes in score of happiness and assertiveness before and after training in two groups

		Steps			

	Group	Before	After	Mean difference	P value
Happiness(Total)	intervention	43.68±17.62	51.57±16.35	7.89	0.049
	Control	44.25 ±12.39	41.74 ±12.21	-2.51		
well-being	intervention	35.93±18.77	49.26±14.67	13.33	0.02
	Control	37.84±14.93	31.94±14.97	-5.9		
life satisfaction	intervention	40.28±26.10	46.11±21.79	5.83	0.23
	Control	42.44±16.33	41.66±16.24	- 0.78		
Respectability	intervention	42.54±15.22	47.94±17.40	5.40	0.59
	Control	41.07±13.78	42.85±13.01	1.78		
Contentment	intervention	48.15±25.55	55.56±26.14	7.41	0.09
	Control	47.91±20.97	41.66±26.13	- 6.25		
Assertiveness (Total)	intervention	110.33±16.05	90.40±12.84	-19.93	<0.0001
	Control	107.68±16.71	106.93±15.81	-0.75		

## 5. Discussion

In this study group assertiveness training effectively increased the level of assertiveness and happiness that it was consistent with the findings of Paeezy et al(Paeezy, Shahraray, & Abdi, 2010), Eroğul (Çeçen-Eroğul & Zengel, 2009), Vatankhah (Vatankhah, Daryabari, Ghadami, & Naderifar, 2013), Mahmoud (Mahmoud & Hamid, 2013), Karagozoglu (Karagözoğlu, Kahve, Koç, & Adamişoğlu, 2008) and Kashani (Kashani & Bayat, 2010) that showed group assertiveness training increase the level of assertiveness. In Paeezy’s study which was conducted on middle school girls, the effectiveness of assertiveness training was evaluated (Paeezy et al., 2010).Results showed that this intervention significantly increase assertiveness scores and academic achievement of students. Also Eroğul (2009), Karagozoglu (2008), Mahmoud (2013) and Kashani (2010) and Vatankhah (2013) showed the effectiveness of group assertiveness training on the self-esteem and self-concept (Çeçen-Eroğul & Zengel, 2009; Karagözoğlu et al., 2008; Kashani & Bayat, 2010; Mahmoud & Hamid, 2013; Vatankhah et al., 2013). Although the results of these studies should compare cautiously with our study, because differ in range of age and gender of subjects. Also Parental substance abuse was not considered as an effective variable in any of the cited studies. This factor can be influential on the educability of these adolescents (Ashenberg Straussner & Huff Fewell, 2012). Adolescents with substance-abusing parents may have poor ability to learning and may not complete academically (McGrath et al., 1999; Wilson, Nunes, Greenwald, & Weissman, 2004).

Results from our study indicated that assertiveness skills training increase the happiness level of subject. These results were in line with studies by Argyle (1990), Shayan (2012), Gilaninia (2012) and Mohammadkhani (2011) (Michael Argyle & Lu, 1990; Gilaninia, 2012; Mohammadkhani & Hahtami, 2011; Shayan & AhmadiGatab, 2012). Argyle (1990) showed that training social skills (including self-expression, assertiveness, stress management, and anger control) can bring about increased levels of happiness (Michael Argyle & Lu, 1990). Shayan et al. displayed the effectiveness of social skills training on happiness levels in Iranian university students. Although intervened group have older age beside our sample and was included both males and females (Shayan & AhmadiGatab, 2012). Gilaninia’s study indicated that training assertiveness skills can cause an increase in the quality of life, emotional adjustment and happiness in students (Gilaninia, 2012). Although in above-mentioned studies disregarded affective factors, such as substance abuse.

A little study has reported positive outcomes such as greater resiliency among adolescents with addicted parents. For example, Gavriel-Fried and Teichman showed that adolescent with substance abuser parents may have a greater sense of ego identity and concretize than peers with not addicted parents (Gavriel-Fried & Teichman, 2007). It has been hypothesized that it may encourage them in developing coping strategies and skills that protect them against experiencing negative effects, such as substance abuse, but this linkage has not been proved (Wolin & Wolin, 1993).

All participants in our study lived in rural areas. Rural youth adult are more likely to use alcohol and other drugs, largely and do dangerous behaviors associated with this use (I. C. Rhew et al., 2011). Due to decline of agricultural over the past few years, rural residents have experienced negative economic consequences. Many rural residents are self-employed and have low income and accordingly the provision of necessary and appropriate medical and mental health services for education or treatment is difficult. In addition, contextual factors, geographic aggregation of socioeconomic privation and deficiency of community and social supplies in rural communities can complicate substance abuse treatment (I. C. Rhew et al., 2011). Also, rural social norms may interfere to utilization of services, as rural residents are more likely than urban residents that because of social stigma don’t use of mental health services (Beardsley, Wish, Fitzelle, O’Grady, & Arria, 2003; Borders & Booth, 2007; I. C. Rhew et al., 2011).

Substance abuse in family membership may be underground, because the stigma of having a substance-abusing parent may elicit some of adolescents that avoid discussing about problems related to this issue with a professional. Then it has been recommended that health care providers should screen all adolescents about substance use difficulties within their families (Werner, Joffe, & Graham, 1999).

To assessing and intervening for children with addicted parents, it is important to consider that each child is unique and has strengths and weakness that should be identified and in each instance reinforced or dissolved. Also, child’s age is an important factor to exerting and selection of intervention that is effective. It means that what is appropriate for a preschool child is not effective the same as that are for an adolescent or a young adult (Ashenberg Straussner & Huff Fewell, 2012).

Also, society supports as teachers, extended family, religious institutions, can be contributing.

Aside the used approach, the main goal of treatment is to improve adolescents’ abilities for care of themselves into emotional, physical, and social aspects. Relieving risk factors and stabilizing resilience are additional goals during the treatment. The clinician should encourage the adolescent to express the feel related to parental substance abuse and its effect on them.

Since 1990, the investigations have found that studies about risk factors related to happiness have different or even inconsistent results. It is because research have utilized different techniques and tools, to excuse other related factors to happiness and not implement causality studies. Dolan indicated that factors such as disease, unemployment, divorce and lack of relationship are negatively related to happiness (Dolan, Peasgood, & White, 2008).

The present study had several limitations. One of the limitations of this study is that our subject contained 57 students in mid-sized rural from one state. Thus, findings from this study may not be nationally representative. Thus, further studies recommend conducting in other settings to examining the effectiveness of group assertiveness training on happiness by rural residential contexts in other large samples for understanding whether these findings are generalized to other area and polities. Second limitation is that limited variables have been considered in our study, For example, factors such as Severity of substance use, household income and personality trait were not assessed in this study. Another limitation is that the girls may get other training program during this study period by different lines such as mass media. Finally, the follow up of the adolescents after the training program was limited in short term. Therefore, a long term follow-up may be more effective.

## 6. Conclusion

Children of substance abusing parents experience higher levels of stress and depression compared to normal individuals in the society. Inappropriate environment, poor parenting, and personality traits inherited from substance abusing parents bring about lower levels of happiness and life satisfaction in these children compared to others (Figlie et al., 2004). Parental substance abuse is a strong predictor of early drug abuse in children (Ashenberg Straussner, 2012) Social skills training programs can reduce the risk of drug abuse in this high-risk group of adolescents by enhancing their life skills. A number of studies on rural girls have shown that this group is at higher risk of developing drug dependency. studies on Iranian rural girls with drug dependent parents showed that these girls’ desire lead them to break free of their family environment (Hojjat, 2013). Poor social skills alongside the social stigma of their parents’ substance abuse have caused these girls to show not resistance against unwanted marriages. Many of these girls believe that they are destined to marry someone they are not interested in, a belief marked by substance abuse in their family members (Hojjat et al., 2013). A kind of tragedy can be seen in their thinking pattern, a tragedy that fate has destined for them. This tragedy starts with their

parents’ substance abuse, leading to unwanted marriages followed by depression and unhappiness, and ends with the struggle to reduce their pains and sorrows through substance abuse (Hojjat et al., 2013). This kind of tragedies could be explain based on learning from the model substance-dependent parents set, children learn to use substance as coping strategies in stressful and difficult times(Otten, Van Der Zwaluw, Van Der Vorst, & Engels, 2008). Training assertiveness skills in rural girls with substance abusing parents can be effective in their decision makings regarding marriage and also prevent drug abuse in future. However, it must be noted that the effectiveness of interventions in this case calls for further study on these rural girls.
